# Recombinant Immunotoxin Therapy of Glioblastoma: Smart Design, Key Findings, and Specific Challenges

**DOI:** 10.1155/2017/7929286

**Published:** 2017-06-29

**Authors:** Shaowei Zhu, Yuanyi Liu, Paul C. Wang, Xinbin Gu, Liang Shan

**Affiliations:** ^1^Molecular Imaging Laboratory, Department of Radiology, College of Medicine, Howard University, Washington, DC, USA; ^2^Department of Neurology, Qilu Hospital of Shandong University, Shandong Province, China; ^3^Angimmune LLC, Rockville, MD, USA; ^4^College of Science Engineering, Fu Jen Catholic University, New Taipei City, Taiwan; ^5^College of Dentistry, Howard University, Washington, DC, USA

## Abstract

Recombinant immunotoxins (RITs) refer to a group of recombinant protein-based therapeutics, which consists of two components: an antibody variable fragment or a specific ligand that allows RITs to bind specifically to target cells and an engineered toxin fragment that kills the target cells upon internalization. To date, over 1,000 RITs have been generated and significant success has been achieved in the therapy of hematological malignancies. However, the immunogenicity and off-target toxicities of RITs remain as significant barriers for their application to solid tumor therapy. A group of RITs have also been generated for the treatment of glioblastoma multiforme, and some have demonstrated evidence of tumor response and an acceptable profile of toxicity and safety in early clinical trials. Different from other solid tumors, how to efficiently deliver the RITs to intracranial tumors is more critical and needs to be solved urgently. In this article, we first review the design and expression of RITs, then summarize the key findings in the preclinical and clinical development of RIT therapy of glioblastoma multiforme, and lastly discuss the specific issues that still remain to forward RIT therapy to clinical practice.

## 1. Introduction

Glioblastoma multiforme (GBM), also known as grade IV astrocytoma, is the most aggressive type of brain tumor. According to the American Brain Tumor Association, GBM accounts for 14.9% of all primary brain tumors and 55.4% of all gliomas in adults, and 12,390 new cases are predicted in 2017 [1]. Despite aggressive and multimodal therapy, the 5-year survival rate is only about 4%. Furthermore, the aggressive nature of current therapies often leads to severe, long-term side effects such as cerebellar mutism, cognitive and endocrine impairments, hearing loss, infertility, and neuropathies. There is a strong need for developing therapies that are more effective in treating GBM, but less toxic to normal brain tissue [2–4].

Recombinant immunotoxins (RITs) represent a promising modality for GBM therapy because of its superior features over monoclonal antibodies (mAbs) and traditional chemotherapeutics [5–7]. First, RITs are much smaller in molecular size, which makes it more efficient to penetrate into solid tumors than mAbs. Second, RITs maintain the specificity of mAbs, but unlike mAbs they are extremely potent and have no known mechanisms of drug resistance. Third, RITs can effectively kill quiescent, nondividing cells, different from traditional chemotherapeutics. Lastly, RITs have little cross-resistance with other agents and are also effective in treating chemorefractory cancer. Since the first report on generation of variable domain fragments of mAbs in 1988, over 1,000 RITs have been generated and RIT development is becoming one of the most fast-growing fields in recent years [8–10]. A large group of RITs have also been generated for GBM therapy and several RITs have entered clinical trials. However, several issues persist as significant barriers to achieving effective therapy.

## 2. Smart Design and Expression of RITs

Immunotoxins have been designed by taking advantage of mAbs or endogenous specific ligands and protein toxins. MAbs and ligands are known to be the most specific agents to an antigen or protein expressed on cancer cells, while toxins are the most potent agent killing cancer cells. Development of immunotoxins evolves over time and technology and can be divided into three generations [11–13]. The first generation was generated by coupling a native, glycosylated toxin with a mAb or a ligand through a cross-linking reagent that forms disulfide bonds between toxin and mAb or ligand. A critical issue for the first generation is the nonspecific binding of the toxin part to normal cells, which not only compromises the specificity of immunotoxins but also causes severe systemic side effects. Accordingly, the binding domain of toxins was deleted when generating the second-generation immunotoxins. This approach significantly reduced the side effects of immunotoxins, but several issues limited their usefulness, including (1) poor stability due to the chemical cross-linking between mAb or ligand and toxin; (2) heterogeneous composition and reduced binding affinity by random conjugation; (3) poor penetration capability because of the large molecular size; (4) strong immunogenicity and off-target toxicity; and (5) limited production [14, 15].

Development of RITs, also called third-generation immunotoxins, is driven by the ability to genetically design and express antibody and toxin fragments with recombinant DNA and protein engineering techniques. As discussed below, the engineered toxin components in the majority of the RITs are from either* Pseudomonas* exotoxin A (PE) or* diphtheria* toxin (DT) [16, 17]. To minimize the immunogenicity induced by PE and DT components, human endogenous cytotoxic enzymes such as RNase, granzyme B, and death-associated protein kinase 2 have also been used in some RITs [18, 19]. These RITs are also called fourth-generation immunotoxins by some investigators. However, the activity of these human endogenous enzymes is much lower than that of PE and DT, resulting in a very low antitumor efficacy.

### 2.1. Design of the Antibody Fragments (Figure 1)

**Figure 1:**
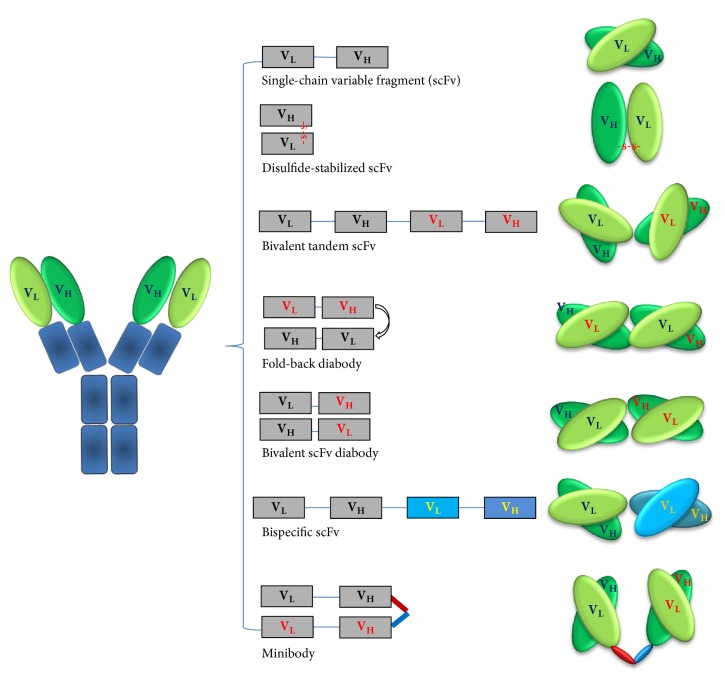
Design of the antibody fragments for construction of recombinant immunotoxins. The left panel shows the “Y”-shape structure of IgG monoclonal antibodies, and the middle and right panels demonstrate the linear and cartoon structures of various antibody fragments. V_H_, the heavy variable region; V_L_, the light variable region.

RITs are constructed using either a specific ligand or an antibody fragment for specific binding to targets [20, 21]. Since most ligands such as transforming growth factor *α* (TGF*α*), epidermal growth factor (EGF), and transferrin are endogenous molecules, they bind to the targets that are not only overexpressed on cancer cells but also physiologically expressed on normal cells. Genetic manipulation of these endogenous ligands often extremely lowers their binding affinity. On the other hand, the candidate targets for most mAbs are usually overexpressed on cancer cells but are expressed much less on normal cells, and some are tumor-specific [22, 23]. Furthermore, mAbs share a relatively uniform and well-characterized protein structure, which allows easy genetic manipulations [24]. Because of these facts, in recent years, mAbs are more frequently engineered to construct RITs. Herein, we focus on the engineering of mAbs (Figure 1).

MAbs are typically composed of two large heavy chains and two small light chains, presenting a “Y”-shape. The small variable region at the two tips of “Y” allows millions of mAbs to recognize different antigens specifically. Since the binding capability of a mAb requires the heavy and light variable regions (V_H_ and V_L_) to work together, the smallest antibody fragment that retains the original binding specificity is the single-chain variable fragment (scFv) (25–30 kDa) that consists of a V_H_ and a V_L_ domain joined by a peptide linker [25, 26]. This linker is usually 12 to 25 amino acids long and rich in glycine for flexibility and serine or threonine for solubility. Accordingly, the smallest RITs, usually called monovalent RITs, are those containing one scFv. Due to the small size (~60 kDa), monovalent RITs exhibit a good penetration capability into solid tumors but suffer from a low binding affinity due to the monovalency. Monovalent RITs are also cleared quickly from the bloodstream (*t*_1/2_=  ~20 min). More desirable pharmacokinetics has been achieved by constructing RITs with a bivalent scFv or scFv-fusion proteins (minibody, 80 kDa; scFv-Fc, 105 kDa) (29-30) [27, 28]. Bivalent scFv refers to a structure with two scFvs that are fused through a peptide linker, which can be designed in two different formats: one is a bivalent tandem scFv (biscFv, 50–60 kDa) when the two scFvs form a single peptide chain, and another is a diabody (50–60 kDa) that is generated by preventing dimerization of the adjacent V_H_ and V_L_ domains from one scFv through a short linker (about five amino acids), while forcing the two scFvs to dimerize by using a long linker (about 15 amino acids) [29, 30]. Bivalent RITs have a binding affinity close to full mAbs. Under most conditions for bivalent binding, two measurable equilibrium dissociation constants (*K*_d_) exist: one for monovalent and the other for bivalent binding. The overall binding affinity of a bivalent scFv is determined by the fraction of bivalent binding. Increasing the bivalent binding fraction is one approach to enhance the binding affinity by optimizing the primary and secondary structures. Kim et al. have compared the binding affinity among different formats and demonstrated that the bivalent fold-back format of RITs is 7-fold and the biscFv format is 2.5-fold higher than the scFv format [28, 29]. Bivalent RITs also have a longer circulation time (*t*_1/2_ =  ~40 min) than monovalent RITs but are still much shorter than antibody-toxin conjugates (*t*_1/2_ ≥ 4–8 hours) [31–33]. Other formats such as triabodies, tetrabodies, and scFv-Fc are used less frequently to construct RITs because the benefit from increased binding affinity could be compromised by the increased molecular size. An alternative format is bispecific tandem scFv that is generated by linking two scFvs from two different mAbs to target different antigens [34–36]. The therapeutic benefit of bispecific RITs is still unclear.

### 2.2. Engineering of Toxin Fragments

The toxin component is typically from either a bacterial protein such as DT and PE or a plant-derived ribosomal inactivating protein like ricin, gelonin, and saporin [37, 38]. Engineered PE or DT fragments are of choice because they are more easily produced with eukaryotic cell systems and induce less side effects than plant toxins [39, 40]. Studies on PE-based RITs are mainly carried out by Dr. Pastan's group at NIH and by several groups in Europe, while studies on DT-based RITs are primarily conducted by Dr. Neville's group at NIH (currently the Angimmune LLC) and by groups in Japan and China.

Both of PE and DT belong to the AB toxin family, consisting of A and B polypeptide chains [41–43]. AB toxins possess three functional domains: one is the receptor binding domain (R domain) that enables toxin to be absorbed on cell surface; one is the translocation domain (T domain) that helps with translocation of A chain into cytosol; and the other is the catalytic domain (C domain) that exerts cytotoxic effects on cells upon translocation to cytosol. DT and PE share a similar cell-killing mechanism and the enzymatic nature allows for extremely high efficiency in killing cells. It is estimated that one single toxin molecule can inactivate over 200 ribosomes or elongation factor 2 molecules per minute and is potent enough to kill a cancer cell, whereas, for a traditional chemotherapeutic drug, it requires 10^4^–10^5^ molecules to reach similar potency [5, 44].

DT protein consists of 535 amino acids. To construct RITs, the R domain has been deleted, which results in toxin fragments with different numbers of amino acids such as DT385, DT388, DT390, DAB389, and DAB486 [45, 46]. Another modification of DT involves the substitution of two amino acids in the B chain, creating a molecule named cross-reacting material 107 (CRM 107) [47, 48]. These modified DT fragments are unable to enter a cell and exert cytotoxic effect themselves. Placement of an antibody fragment or a ligand to the C-terminal of a DT fragment has much less adverse influence on the DT activity than placement to its N-terminal. To reduce the immunogenicity of DT fragments, Schmoh et al. have recently generated several mutated forms of the DT390 by mutating the highly hydrophilic R, K, D, E, and Q amino acids on the DT390 molecular surface [49]. Animal studies have demonstrated 90% reduction in production of neutralizing antibodies in mice immunized with the mutants. The RITs constructed with the mutant DT390 exhibit only minimal loss of the activity in vitro and in vivo.

PE toxin is a single-chain 66-kDa polypeptide. Proteolytic cleavage of PE in endocytic vesicles occurs near arginine-279, generating a 37 kDa fragment that is translocated to cytosol. There are several other protease-sensitive sites that are considered to be not essential for PE activation. Deleting its cell-binding domain results in different sizes of fragments such as PE35 (35 kDa), PE38 (38 kDa), and PE40 (kDa) [50–53]. In recent years, two modified PE38 fragments, PE38KDEL and PE38QQR, are more frequently used in the generation of PE-based RITs. PE38KDEL carries a C-terminal with the last four amino acids of KDEL (lysine, aspartic acid, glutamic acid, and leucine). For PE38QQR, the domain Ia (amino acids 1–252) and amino acids 365–380 of PE are deleted, and lysine residues at positions 590 and 606 are replaced with glutamine and at 613 replaced with arginine. The two fragments exhibit improved intracellular retention and reduced hepatotoxicity. To reduce the immunogenicity of PE fragments, Dr. Pastan's laboratory has mapped the T- and B-cell epitopes of PE38 fragments and eliminated these epitopes by mutagenesis [51–53]. The resulting PE fragments have been shown to induce much less immunogenicity but maintain their enzymatic activity. PE is relatively resistant to genetic manipulation over DT.

### 2.3. Expression Systems of RITs

RITs are expressed mainly using yeast, bacteria, or cell expression systems [54, 55]. Each system has its unique features, but, as an expression system, two critical requirements must be met: (1) capability of properly folding complex proteins with multiple domains and (2) resistance to the toxin moiety. Being inexpensive, being fast, and being easy to produce and purify are other requirements. Bacterial systems like* E. coli* are resistant to DT and PE toxins and are easy to manipulate; thus they are widely used to express RITs. A major disadvantage is that bacterial systems lack the ability to efficiently fold complex proteins. RITs must be denatured and refolded ex vivo to recover their binding capability and bioactivity. Unfortunately, the recovery is often incomplete. It is difficult to produce multidomain RITs with high activity using bacterial systems. Toxin-resistant mammalian cell lines such as CHO and HEK293T cells are also used to produce RITs, but it is labor-intensive and time-consuming to select and characterize toxin-resistant cell lines [56]. Limited production yield and complicated purification are among other issues for using mammalian cell lines. Yeasts like* Pichia pastoris (P. pastoris)* could grow in a simple, inexpensive medium with a high growth rate in either a shake flask or a fermenter, making them suitable for both small- and large-scale production. Importantly,* P. pastoris* itself is capable of properly folding RITs by producing disulfide bonds (57–59). Similar to mammalian cell lines, yeasts are sensitive to toxins and they are essential to select toxin-resistant strains.

## 3. Key Findings of Preclinical and Clinical Studies on RIT Therapy of GBM (Tables 1 and 2)

Both PE- and DT-based RITs are known to directly kill targeted cells by inhibiting cell protein synthesis through ADP-ribosylation of the elongation factor 2. Recent studies further demonstrate that RIT induces delayed cytotoxic effects and tumor regression [57, 58]. In some patients, tumor regression occurs after a period of RIT withdrawal, following suboptimal drug delivery, or when heterogeneous expression of the targeted antigen exists within tumors. Immune responses induced by released antigens following cell killing have been hypothesized as the secondary antitumor mechanism of RITs. Studies by Ochiai et al. have revealed that the antitumor response is induced after intratumor injection of an EGF receptor (EGFR) variant III- (EGFRvIII-) specific RIT, but this response is reduced when CD4^+^ and CD8^+^ T-cells are depleted [57]. The investigators have further observed that the tumor cells without EGFRvIII expression are similarly eliminated [59]. The latter finding is explained by cross-presentation of antigens subsequent to the killing of EGFRvIII-expressing tumor cells. Below are some key findings in the preclinical and clinical development of RITs for GBM therapy.

### 3.1. RITs Targeting EGFR and EGFRvIII

EGFR is a transmembrane tyrosine kinase belonging to the HER/erbB family. To date, at least seven peptide ligands have been documented for EGFR including EGF and TGF*α*. Binding with EGFR leads to internalization of both ligands and receptor and trafficking to early endosomal compartment of the cells [124–126]. EGFRvIII is a tumor-specific mutation of EGFR, which is expressed highly in various types of cancer [127–129]. EGFRvIII results from an in-frame deletion of exons 2–7 of the EGFR gene. This deletion, together with insertion of a glycine residue, produces a unique junctional peptide at the deletion interface. Approximately 60–90% of GBM overexpress and 40–50% have amplified EGFR, and up to 60–70% of the EGFR-amplified GBM possess EGFRvIII [3, 4]. The high prevalence of EGFR/EGFRvIII overexpression as well as the tumor specificity of EGFRvIII makes EGFR/EGFRvIII attractive targets for generation of RITs [130, 131].

#### 3.1.1. D2C7(scdsFv)-PE38KDEL (D2C7-IT)

D2C7-IT is a monovalent RIT generated by fusing a disulfide-stabilized scFv from the D2C7 antibody with the PE38KDEL fragment [60, 61]. The D2C7 antibody was generated by immunizing mice with a peptide corresponding to the junction created by EGFRvIII. D2C7 recognizes both wild-type EGFR and EGFRvIII proteins. In vitro, D2C7-IT demonstrates high cytotoxicity to GBM cell lines. The half maximal inhibitory concentration (IC_50_) of D2C7-IT was reported to be 0.18 to 2.5 ng/mL against cells overexpressing wild-type EGFR (NR6W, A431, 43, and D08-0493MG cells), and approximately 0.25 ng/mL against cells expressing EGFRvIII (NR6M cells) or coexpressing EGFR/EGFRvIII (D2159MG and D270MG cells). In the rat intracranial models of 43, NR6M, and D270MG tumor xenografts, convection-enhanced delivery (CED) of D2C7-IT has prolonged survival of rats by 310%, 28%, and 166%, respectively, compared to control rats. D2C7-IT exhibits minimal binding to nontumor brain tissues [62]. Preclinical toxicity evaluation following CED of D2C7-IT to Sprague-Dawley rats has revealed a maximum tolerated dose (MTD) of 0.10–0.35 *μ*g and a no-observed-adverse-effect-level of 0.05 *μ*g [63].

D2C7-IT is currently in Phase I/II study to determine its MTD and initial effectiveness in patients with advanced GBM (NCT02303678). The final results are still pending. 

#### 3.1.2. MR1(Fv)-PE38 and MR1-1(Fv)-PE38

MR1(Fv)-PE38 is constructed by fusing MR1(Fv) with PE38 fragment for treatment of GBM expressing EGFRvIII [64–67]. MR1(Fv) is a scFv that was isolated from a library of phage displaying murine scFv. MR1-1(Fv) is a mutated version of MR1(Fv), which was generated through targeted mutagenesis of the complementary determining region 3 (CDR3) of the heavy and light chains of MR1(Fv). The sequence of MR1-1(Fv) differs from MR1(Fv) by three amino acids in the V_H_ and V_L_ CDR3 domains. Binding studies have shown that MR1-1(Fv) has a 15-fold higher affinity with EGFRvIII than MR1(Fv). *K*_d_ was measured to be 1.5 × 10^−9 ^M for MR1-1(Fv) versus 2.3 × 10^−8 ^M for MR1(Fv) [66]. In biodistribution studies using athymic nude mice bearing subcutaneous EGFRvIII-expressing U87 tumor xenografts, an up to 244 ± 77% increase in tumor uptake for MR1-1(Fv)-PE38 was observed compared with that for MR1(Fv)-PE38 [67]. In rat models of GBM, MR1-1(Fv)-PE38, when delivered directly to tumor tissue, displayed a 3.5-fold increased potency towards cells expressing EGFRvIII, compared to MR1(Fv)-PE38. MR1-1(Fv)-PE38 extended animal median survival to more than 53 days, compared to control animal survival of seven days. All animals survived the treatment with no signs of neurotoxicity or other noticeable adverse effects.

Ochiai et al. have analyzed the antitumor efficacy of MR1-1(Fv)-PE38 in the immunocompetent mice bearing subcutaneous SMA560msEGFRvIII tumor xenografts [57]. SMA560 is a malignant astrocytoma cell line and SMA560msEGFRvIII is the SMA560 cell line stably transfected with a mouse homologue of EGFRvIII. Intratumoral administration of MR1-1(Fv)-PE38 eliminated the EGFRvIII-expressing tumors. Interestingly, the antitumor activity was observed to be dependent on the expression of EGFRvIII on some, but not all tumor cells, and the activity could be significantly inhibited in the absence of CD4^+^ and CD8^+^ T-cells [57]. The investigators conclude that MR1-1(Fv)-PE38 induces EGFRvIII-specific immunity and produces long-lasting immunity against tumor cells expressing EGFRvIII as well as those without expression of EGFRvIII.

MR1-1(Fv)-PE38 entered Phase I trial for safety profiling in treatment of malignant brain tumors; however this trial has been terminated due to low accrual [123].

#### 3.1.3. TGF*α*-PE38 (TP38) and TGF*α*-PE40 (TP40)

TGF*α* is a mitogenic polypeptide that belongs to the EGF family. TGF*α* acts as either a transmembrane-bound ligand or a soluble ligand with similar biological functions to EGF. TGF*α* has been fused with different PE fragments, resulting in several RIT variants such as TP40, TP38, TP35, and TP31 [68–70]. Of them, TP38 has been evaluated for its effects more extensively. In an intracranial brain tumor model, epidermoid carcinoma A431 cells were mixed with TP38 and implanted into the caudate nuclei of athymic mice; mice receiving tumor cells mixed with either 0.03 *μ*g or 0.1 *μ*g of TP38 displayed 90% and 100% survival compared to mice injected with cells alone (19 days) [71]. Toxicity assessment revealed a MTD of 0.66 *μ*g when directly injected into the caudate nucleus of athymic rats. The rats treated with high dose of TP38 exhibited demyelination and necrosis. When TP38 was infused into the brain of rhesus macaques, the MTD was determined to be 6 *μ*g [71].

Sampson et al. have reported the findings of Phase I clinical trial of TP38 in 20 adult human patients with recurrent brain tumors [58, 59]. By direct infusion of TP38 into the brain, the most toxicities encountered were solely neurological and most likely unrelated to TP38, rather a consequence of infusion volume, recurrent tumor, or stereotactic catheter placement. In this study, the dose escalation of TP38 was stopped at 4 *μ*g without reaching its MTD due to inconsistent drug delivery [120, 121]. The median time to tumor progression was 14.9 weeks, and the median survival was 28 weeks in these patients. The investigators noticed that many patients experienced significant leaks of the drug into the ventricles or subarachnoid space, resulting in failed intraparenchymal distribution, although the treatment was considered to be safe. In another dose escalation study, MTD has also not been established. The overall median survival for all patients was 23 weeks, and, for those without radiographic evidence of residual disease at the time of therapy, the median survival was 31.9 weeks [59]. Two dose-limiting neurologic toxicities were observed, including grade 3 hemiparesis and grade 4 fatigue. Again, more than 80% of infusions resulted in drug leakage to other areas of brain.

TP40 has been analyzed for its cytotoxicity in cell lines and in Phase I clinical trial as an intravesical therapy [72–74, 132]. The trial showed that TP40 was well-tolerated in patients with superficial bladder cancer with no dose-limiting toxicities between 0.15 mg/week and 9.6 mg/week. Eight of nine patients with carcinoma in situ demonstrated partial or complete responses to treatment. Patients with invasive disease showed no response to the treatment and no visible changes were observed in tumors. In general, Phase I study for early superficial bladder cancer is encouraging. Phase II studies have not been initiated. No clinical studies on brain tumors have been reported with TP40.

#### 3.1.4. DAB389EGF

DAB389EGF is a fusion protein of EGF and DT389 fragments [75, 76]. DAB389EGF demonstrates potent cell-killing ability at pM concentrations against a panel of human GBM cell lines [76, 77]. The efficacy of DAB389EGF is strongly correlated with the EGFR density on GBM target cell lines. In animal models, the MTD of DAB389EGF by intratumoral injection of subcutaneous U87 tumors every other day for three to six doses was measured to be 3 *μ*g [78–80]. At this dosage, tumor regression was observed in all animals; however 25% of the animals exhibited a tumor relapse within one month. Relapsed tumors were found to retain their EGFR and responded to a second round of treatment. Animals receiving higher doses exhibited weight loss, diminished activity, and dehydration. Altered blood chemistry included urea nitrogen, creatinine, aspartate transaminase, and alanine transaminase. Histopathological analysis of kidney revealed renal tubular necrosis.

Phase I/II clinical trial has been conducted in 52 patients with metastatic diseases [122]. One patient with non-small-cell lung carcinoma displayed a partial response, and three others showed stable disease through the duration of the trial. However, all patients developed anti-DT neutralizing antibodies. The adverse effects included fever, malaise, nausea/vomiting, hypoalbuminemia, hypertension, and anorexia. One patient experienced proximal renal tubular acidosis. Dose-limiting toxicity was determined to be 9 *μ*g/kg/day for five consecutive days and 15 *μ*g/kg/day in an episodic dosing regimen on days 1, 8, 9, 15, and 16 every 28 days.

#### 3.1.5. DT390-BiscFv806

DT390-BiscFv806 is a bivalent RIT generated in our laboratory [81]. This RIT is designed by taking advantage of the unique specificity of mAb806 to the EGFR and EGFRvIII overexpressed in cancer [81]. The mAb806 was raised against mouse fibroblast cells expressing EGFRvIII. The mAb806 binds to an epitope exposed only in the transitional untethered form of EGFR when it is overexpressed in cancer [133–137]. We first generated a bivalent RIT, designated as DT390-MuBiscFv, by fusing DT390 with a biscFv from the murine mAb806 through peptide linkers ((G_4_S)_3_). Use of biscFv significantly improves the binding affinity of RITs, ~2.5-fold higher than that of monovalent version. Encouraged by the promising results and leveraging the mAb806 humanization-derived benefits, we further generated a humanized biscFv RIT, designated as DT390-HuBiscFv. Both bivalent RITs were expressed using a DT-resistant* P. pastoris* system invented by our collaborators (Patent number US7892786). DT390-HuBiscFv maintains the specificity of mAb806 and is extremely cytotoxic to various cancer cell lines with EGFRvIII. DT390-HuBiscFv shows two to three orders of magnitude more potent to the EGFRvIII-transfected U87 (IC_50_, 1 × 10^−13 ^M) than to the parental U87 cells (IC_50_, 8 × 10^−10 ^M). Systemic administration of DT390-HuBiscFv inhibited the growth of established tumor xenografts produced by the U87 cancer cells with and without EGFRvIII, showing an inhibition rate of 76.3% (59.82–96.2%) and 59.4% (31.5%–76.0%), respectively [81].

#### 3.1.6. ScFv (14E1)-ETA

ScFv (14E1)-ETA is generated by fusing the scFv from the mAb 14E1 with PE40 [82, 83]. 14E1 was isolated from mice immunized with A431 epidermoid carcinoma cells and recognizes both wild-type EGFR and EGFRvIII. In vitro, scFv (14E1)-ETA exhibits similar potency to TP38 against multiple cancer cell lines with EGFR overexpression, but, unlike TP38, scFv (14E1)-ETA also binds EGFRvIII, displaying 100-fold more potency towards cells with EGFRvIII expression than cells with wild-type EGFR expression only [82, 83]. Coadministration of cisplatin and scFv (14E1)-ETA shows a synergistic effect on killing the chemotherapy-resistant cells, more effective than either treatment alone [84]. In immunocompetent mouse models, scFv (14E1)-ETA also demonstrates antitumor capabilities; however all mice developed anti-PE neutralizing antibodies, resulting in neutralization of toxin activity [85].

#### 3.1.7. Anti-EGFR/LP1

Anti-EGFR/LP1 is a fusion protein of a 5 kDa ribosome-inactivating polypeptide Luffin P1 (LP1) and an anti-EGFR scFv connected via a (GGGGS)_3_ flexible polypeptide [86]. LP1 is the smallest type III plant ribosome-inactivating proteins (RIPs) [138]. Similar to most RIPs, LP1 inhibits protein synthesis by depurinating the large ribosomal RNA, thereby blocking ribosome binding to elongation factor 2 [139]. Anti-EGFR/LP1 was expressed in* E. coli*, refolded, and purified on an immobilized Ni^2+^-affinity chromatography column. Anti-EGFR/LP1 displays growth inhibition of EGFR-expressing U251 cells [86]. Its efficacy and toxicity in animal models of GBM are unclear.

### 3.2. RITs Targeting Interleukin- (IL-) 4 and IL-13 Receptors

IL-4 and IL-13 are two cytokines related closely in both structure and function [140, 141]. The IL-4 and IL-13 genes are both mapped on chromosome 5q. The effect of IL-4 signaling is mediated through the IL-4 receptor *α*-chain (IL-4R*α*) [142]. Upon binding to IL-4, IL-4R*α* dimerizes either with the common *γ*-chain to produce the type 1 signaling complex or with the IL-13 receptor *α*-chain 1 (IL-13R*α*1) to produce the type 2 complex [143]. IL-13 has two receptors: IL-13R*α*1 and IL-13R*α*2. IL-4R*α* and IL13R*α*1 are expressed ubiquitously in various tissues including normal brain tissue [144, 145]. Under physiological conditions, they regulate immune response and immune microenvironment. In various types of cancer including GBM, IL-4R*α*, and IL-13R*α*1, they have been shown to be overexpressed and promote tumor proliferation, cell survival, and metastasis [140, 141]. Different from IL-13R*α*1, IL-13R*α*2 has a very high binding affinity with IL-13 but not with IL-4. IL-13R*α*2 is selectively expressed on glioma cells and associated with increased malignant grade and poor patient prognosis [146, 147]. Because IL-13R*α*2 does not express or express little in normal brain tissue, IL-13R*α*2 appears more attractive than IL-4R*α* and IL-13R*α*1 as a target for RIT generation.

#### 3.2.1. IL-13PE38QQR (IL-13PE), IL13E13K-PE38, and Anti-IL-13R*α*2(scFv)-PE38

IL-13PE is made by fusing human IL-13 and PE38QQR [87–89]. In vitro, IL-13PE demonstrates high cytotoxicity to IL-13R-positive tumor cell lines including GBM, AIDS-associated Kaposi's sarcoma, and cancer arising from kidney, head and neck, ovary, prostate, colon, and skin. The cytotoxicity is correlated with the number of receptor sites on tumor cell surface. Systemic administration is limited by dose due to toxic side effects. Local administration allows IL-13PE active for approximately six hours at the site of injection. The antitumor efficacy following intratumoral administration has been demonstrated in a number of tumor xenograft models and shown evidence of activating the innate immune response that mediates robust tumor response [148]. IL-13PE is more cytotoxic to the tumors that preferentially express high level of IL-13R*α*2.

For the IL13E13K-PE38, IL-13 is replaced with its mutated form, IL-13E13K, in which glutamic acid (E) residue at position 13 of IL-13 molecule is substituted by a lysine (K) [88, 92]. IL-13E13K exhibits a higher affinity to IL-13R than the wild-type IL-13 [90]. It has been shown that the affinity of IL13E13K-PE38 to U251MG and IL-13Ra2 chain-transfected tumor cell lines is 3 to 10 times higher than that of IL-13PE. However, they have similar cytotoxicity [91, 92]. The antitumor activity of IL13E13K-PE38, when administered intraperitoneally to nude mice bearing U251 tumors, is also similar to that of IL-13PE. Some improvement in antitumor activity has been observed only when lower doses of IL13E13K-PE38 are injected into tumors. In general, IL13E13K-PE38 mediates similar cytotoxicity and antitumor activity to IL-13PE, despite its improved binding affinity to IL-13R.

In several studies, IL-13PE has been integrated into adenoviral vectors to express IL-13PE along with the virus replication [71, 149]. When injected into tumors, the adenoviral vectors encoding IL-13PE have been shown to provide long-term and high local expression of IL-13PE and lead to an effective cytotoxic response in IL-13R*α*2-expressing GBM cells with less side effects to the surrounding normal brain tissue. A single intratumoral injection of such a therapeutic vector into intracranial human GBM xenografts and murine GL26 tumors in immunocompetent mice resulted in tumor regression and long-term survival in 50–70% of the animals [71].

Different from IL-13PE, anti-IL-13R*α*2(scFv)-PE38 is generated with a high-affinity clone of scFv against IL-13R*α*2 [91, 92]. This clone was isolated from a human scFv antibody phage library. The anti-IL-13R*α*2(scFv)-PE38 is highly cytotoxic to U251 glioma and other cancer cell lines in vitro. Its cytotoxic activity can be neutralized by purified extracellular domain of IL-13R*α*2 but not by IL-13, indicating that it is highly specific to IL-13R*α*2. In immunodeficient mice bearing subcutaneous glioma tumors, anti-IL-13R*α*2(scFv)-PE38 demonstrated significant antitumor activity with a MTD of 200 mg/kg when given intraperitoneally twice daily for 5 days. The high specificity of anti-IL-13R*α*2(scFv)-PE38 suggests it has less toxic side effects than the RITs constructed with human IL-13 [92].

Several Phase I/II clinical trials have been conducted in a total of 120 GBM patients to evaluate intracerebral CED of IL-13PE (trade name: Cintredekin Besudotox) [116, 117]. The patients underwent an initial tumor biopsy procedure, followed by the placement of one intratumoral catheter, and IL-13PE was then administered by CED over a period of 48 hours (dose escalation 0.25–2 mg/mL, 400 mL/hour) [117]. In another study, two or three catheters were inserted into the region adjacent to the tumor resection cavity (peritumoral infusion) and IL-13PE (0.25 mg/mL, 750 mL/hour) was then administered over 96 hours [118]. The maximum tolerated intraparenchymal concentration was determined to be 0.5 mg/mL and tumor necrosis was observed at this concentration. Infusion durations of up to 6 days were well-tolerated. The overall median survival duration was 42.7 weeks (95% confidence interval 35.4–59.3) in 42 patients receiving peritumoral infusion (0.25 and 0.5 mg/mL) [118]. The outcomes were found to be better when two or more catheters were adequately positioned (0.25 and 0.5 mg/mL, 57.4 weeks, 95% confidence interval 35.6–75.3, 24 patients) [118]. Intracranial administration led to dose-limiting toxicities in some patients, including neurological symptoms secondary to necrotic and inflammatory processes, irreversible hemiparesis, and the death of one patient due to neurologic decline.

Based on these results, a randomized controlled Phase III clinical trial (PRECISE Trial) has been conducted [119]. This trial enrolled 296 patients; one arm received IL-13PE and another received carmustine-releasing gliadel wafers (GW) via catheters implanted in the walls of the resection cavity after craniotomy. IL-13PE was well-tolerated but showed similar efficacy (overall survival) to GW. Retroactive data analysis of time-to-progression was significantly longer with IL-13PE compared to GW (17.7 versus 11.4 weeks). A follow-up study noted that only 68% of catheter placements were performed per protocol, suggesting that variability in catheter positioning may have adversely impacted results.

#### 3.2.2. IL4(38-37)-PE38KDEL (cpIL4-PE)

cpIL4-PE is a protein comprised of circularly permuted human IL-4 and PE38KDEL [95, 96]. cpIL4-PE has been shown to be highly cytotoxic to glioma cell lines in vitro but not or less to hematopoietic and normal brain cells. IC_50_ was measured to be 1 ng/mL for three of five medulloblastoma cell lines expressing >900 IL-4 binding sites/cell, 30 ng/mL for D341 cell line expressing ~600 sites/cell, and no marked cytotoxicity at concentrations up to 1000 ng/mL for D283 cell line expressing the lowest level of IL-4R [96]. The cytotoxic activity of cpIL4-PE can be neutralized by either excess IL-4 or IL-13, indicating that IL-4R are related to IL-13R on medulloblastoma cell lines. This property of receptor sharing for IL-4 and IL-13 has also been observed in other cancer cell lines. Intratumoral injection of cpIL-4PE in human tumor xenografts including GBM has all shown remarkable antitumor effects.

In an open-label, dose escalation Phase I trial by Weber et al., drug-related grade 3 and 4 central nervous system toxicities were observed in 39% and 22% of patients, respectively [113]. The MTD was determined to be 6 *μ*g/mL in 40 mL. The six-month survival was 48% and the overall median survival was 5.8 months for the GBM patients. The patients with recurrent GBM in the placebo arm of this trial had an overall median survival of 4.7 months and a six-month survival rate of 36%. In another trial, the safety and activity of cpIL4-PE were investigated after directly infusing into gliomas of nine patients over a 4–8-day period by one to three stereotactically placed catheters [114]. Neither apparent systemic toxicity nor histological evidence of neurotoxicity to normal brain was identified in any patients. Local toxicity seemed attributable mainly to tumor necrosis or occasionally to the volume of infusion. A multicenter, randomized, open-label Phase II study is currently ongoing in patients with recurrent GBM to evaluate efficacy of intratumoral administration of cpIL-4PE after surgical resection, with a secondary objective to evaluate safety and tolerability of this immunotoxin (NCT00014677). Currently, there are no Phase III protocols involving cpIL-4PE.

The safety and tolerability of cpIL-4PE have also been demonstrated in an additional Phase I clinical trial for renal cell carcinoma and non-small-cell lung carcinoma [115]. Cohorts of three to six patients were treated at dose levels of 0.008, 0.016, and 0.027 mg/m^2^ daily × 5 days every 28 days. Fourteen patients received 1–6 cycles of cpIL-4PE. No dose-limiting toxicities were noted at dose levels of 0.008 and 0.016 mg/m^2^. At 0.027 mg/m^2^, two patients developed self-limiting, grade 3 or 4 transaminase elevation during treatment cycle. However, no objective tumor responses were noted. Low circulating level of cpIL-4PE, coupled with rising neutralizing antibody titers, may contribute to the lack of response.

#### 3.2.3. DT_390_IL13 and DT390-mIL4

DT_390_IL13 is an IL-13R-targeted RIT, which is composed of a DT390 fragment instead of PE fragment [93, 94]. DT_390_IL13 was found to inhibit the U373MG GBM cell growth with IC_50_ of ~12 pM. In nude mice, small U373MG tumor xenografts completely regressed in most animals after five intratumoral injections of 1 mg of DT_390_IL13 q.o.d. for five doses [93]. DT_390_IL13 has also been tested against primary explant GBM cells of a patient's excised tumor and IC_50_ is similar to that for U373MG. Toxicity studies demonstrate that DT_390_IL13 of 1–30 mg/injection has little effect on kidney, liver, spleen, lung, and heart in immunocompetent mice.

DT390-mIL4 is constructed with DT390 and murine IL-4 [97]. This RIT exhibited a dose-dependent cytotoxic effect with IC_50_ of 0.56 × 10^−9 ^M against SMA-560, 1.28 × 10^−9 ^M against neuro-2a, and 0.95 × 10^−10 ^M against NB41A3 cells. The cytotoxicity of DT390-mIL4 was specifically blocked by excess of anti-mouse IL-4 monoclonal antibody (11B11).

### 3.3. Bispecific RITs

#### 3.3.1. DT390-ATF (DTAT) and DT390-IL-13-ATF (DTAT13)

DTAT is a fusion protein containing DT390 and the noninternalizing amino terminal fragment (ATF) portion of human urokinase-type plasminogen activator (uPA) [98]. The ATF portion lacks the catalytic domain of uPA but possesses an EGF-like or growth factor domain that comprises the receptor binding sequence of uPA. Different from DTAT, DTAT13 is designed to target both the uPA receptor (uPAR) and IL13R*α*2 [99–101]. Accordingly, DTAT13 is generated by adding the ATF of uPA to DT-IL13 with a linear sequence of DT390-IL-13-ATF.

The uPAR is a glycosylphosphatidylinositol-anchored receptor located on the cell surface. Functionally, uPAR regulates extracellular matrix proteolysis, cell-extracellular matrix interactions, and cell signaling [101, 150]. In several types of cancers including GBM, uPAR expression has been reported to be elevated and its expression level is correlated with tumor invasiveness and shorter survival of patients. The endothelial cells of tumor neovasculature also express uPAR. Therefore, uPAR serves a therapeutic target against both tumor cells and neovasculature [150, 151].

In vitro, DTAT is highly potent and selective against U118MG, U87MG, and U373MG GBM cell lines and human umbilical vein endothelial cells (HUVEC) [99]. IC_50_ of DTAT was measured to be 0.24 nM for U87MG cells and 2 nM for HUVEC. DTAT13 has similar cytotoxicity to DTAT, exhibiting IC_50_ of 0.2 nM for U87MG cells and 0.0007 nM for U373MG cells. DTAT13 also inhibits HUVEC growth in a dose-dependent manner. In vivo, both DTAT and DTAT13 resulted in significant regression of subcutaneous U87MG tumors when administered every other day at 10 mg/day for five doses [100]. Liver alanine aminotransferase levels were found to be significantly increased, but not to life-threatening levels. Mortality studies indicate that DTAT13 is less toxic than DTAT, suggesting that it may allow treatment of a broader subset of antigenically diverse tumors with reduced exposure to toxins than if two separate agents were employed.

In the studies by Rustamzadeh et al., the MTD of DTIL13 was measured to be 1 mg/injection every other day for three injections [101]. Doses that exceeded this amount resulted in weight loss and liver damage. The same dose given to nude mice with established intracranial U373MG tumors resulted in prolonged survival and significant reduction in tumor volume. The pharmacokinetic experiments following intracranial injection of radiolabeled DTIL13 showed that DTIL13 was mainly cleared by the kidneys.

#### 3.3.2. EGFATFKDEL and EGFATFKDEL7mut

The two RITs are designed to simultaneously target both the EGFR that are overexpressed on cancer cells and the uPAR on tumor neovasculature [102–104]. EGFATFKDEL consists of human EGF, a fragment of uPA, and PE38KDEL. EGFATFKDEL7mut is a version with reduced immunogenicity constructed by mutating seven immunodominant B-cell epitopes on the PE38KDEL molecule. Both RITs are effective against glioblastoma cell lines as well as HUVEC. In mice with subcutaneous GBM xenografts, intratumoral injection of EGFATFKDEL7mut eradicated small tumors in over half of the treated mice, which then survived with tumor-free status for at least 100 days after tumor inoculation [103]. Immunization experiments in immunocompetent mice revealed significant reduction of anti-toxin antibody production in EGFATFKDEL7mut-treated mice, compared to in EGFATFKDEL-treated mice. Oh et al. tested a similar construct and showed that EGFATFKDEL7mut selectively kills the glioblastoma cell line U87-luc as well as cultured human endothelial cells in vitro [104]. In vivo, when rats bearing brain tumors were treated via CED of the drug, significant tumor reduction was observed and some rats survived with a tumor-free status for 130 days after tumor inoculation. The MTD of EGFATFKDEL7mut was established at 2 *μ*g/injection or 8.0 *μ*g/kg, and this dose was nontoxic. Antitoxin antibodies were reduced by at least 90% [104].

#### 3.3.3. DTEGF13

DTEGF13 is a bispecific RIT that is composed of IL-13, EGF, and DT390 [105, 106]. In vitro, DTEGF13 selectively kills the human glioblastoma cell lines, U87MG (IC_50_, 0.015 nM), and U118MG (IC_50_, 0.02 nM). Interestingly, DTEGF13 exhibits a greater activity than either of its monospecific counterparts or their mixture, proving it necessary to have both ligands on the same single-chain molecule [105]. The cytotoxicity could be blocked with anti-EGFR and anti-IL-13 antibodies. In the subcutaneous tumor xenograft model, intratumoral injection of DTEGF13, but not monospecific DTEGF or DTIL13, significantly inhibited the growth of established U87 tumors in nude mice [105]. In aggressive intracranial tumors established in nude rats with U87 cells, two injections of DTEGF13 via CED resulted in tumor eradication in 50% of the rats, which survived with tumor-free status for at least 110 days after tumor inoculation [106]. The MTD was measured to be 2 mg/injection or 0.5 mg/kg. No anti-DT antibodies were detected in normal immunocompetent rats when given identical intracranial dosage of DTEGF13. Combination of monospecific DTEGF and DTIL13 did not inhibit tumor growth.

### 3.4. RITs Targeting Other Antigens or Receptors

#### 3.4.1. H9scFv-PE38 Targeting B7-H3 (CD276)

8H9scFv-PE38 is a fusion protein consisting of PE38 and a scFv from mAb 8H9 [107, 108]. MAb 8H9 is a murine IgG1 hybridoma derived from the fusion of mouse myeloma SP2/0 cells and splenic lymphocytes of BALB/c mice immunized with human neuroblastoma [152]. This antibody recognizes B7-H3 (CD276) antigen, a type I transmembrane protein with 20–27% amino acid identity with other B7 family members. Functionally, B7-H3 exhibits complex interactions with T-cells and natural killer cells, and its expression is induced on these cells [153]. B7-H3 mRNA is broadly expressed in normal tissues, but its protein expression is relatively rare. Interestingly, B7-H3 protein is expressed in various types of cancer [154]. Immunohistochemistry has shown that the B7-H3 epitope recognized by 8H9 is not expressed by normal neurons or glia but demonstrates immunoreactivity in a vast majority of human GBM and anaplastic astrocytoma samples [152]. In vitro, 8H9scFv-PE38 is cytotoxic against GBM cell lines, having IC_50_ of 1265 ng/mL for U87 and 91 ng/mL for U251. When the 8H9scFv-PE38 was interstitially infused to the striatum and brain stem of rats, its MTD was determined to be 0.75 *μ*g and 1.8 *μ*g, respectively. In rats harboring intracranial U87 xenografts, infusion of 8H9scFv-PE38 has been demonstrated to increase the mean survival (striatum: 43.4 days (treated) versus 24.6 days (placebo); brain stem: 80.6 days (treated) versus 45.5 days (placebo)) [107, 108]. Tumors showed volumetric response to 8H9scFv-PE38 by magnetic resonance imaging.

#### 3.4.2. EphrinA1-PE38QQR Targeting EphA2 Receptor

EphrinA1-PE38QQR is generated by fusing the endogenous EphA2 receptor ligand, Ephrin A1, to PE38QQR [109]. The Eph receptor family is comprised of two subclasses, EphA (EphA1-10) and EphB (EphB1-6) [155]. The first member, named EphA1, was cloned from an erythropoietin-producing hepatocellular cancer cell line and the second member, EphA2, was identified by screening the human epithelial (Hela cells) cDNA library. The ligands for Eph receptor family are also divided into two subclasses: EphrinA (EphrinA1-6) and EphrinB (EphrinB1-3). EphrinA1 was identified as a cytokine-inducible gene product in human HUVEC cells and is a ligand for EphA2 receptor. EphrinA1 is the most extensively studied ligand for EphA2 in cancer, although EphA2 can be activated by other EphrinA ligands in cancer cells and tumor vasculature. EphA2 is found to be overexpressed in several GBM cell lines and is predominantly localized on the cell membrane [156, 157]. In human glioma tissues, EphA2 shows a heterogeneous staining pattern [158]. The normal brain tissues have minimal staining. EphrinA1-PE38QQR exhibits a potent and dose-dependent cytotoxicity to GBM cells, with IC_50_ of approximately 10^−11 ^M. No cytotoxicity is observed to normal human endothelial cells and EphA2^−^ tumor cells [109].

#### 3.4.3. NZ-1-(scdsFv)-PE38KDEL Targeting Podoplanin

NZ-1-(scdsFv)-PE38KDEL is formed by fusing a scFv from the NZ-1 antibody with PE38KDEL [110]. The scFv fragment is stabilized by a disulfide bond between V_H_ and V_L_. NZ-1 is a rat IgG_2a_ antibody, recognizing human podoplanin [159]. Podoplanin is a 162-amino acid type I transmembrane sialomucin-like glycoprotein, consisting of a serine- and threonine-rich extracellular domain [160]. Podoplanin is expressed in several types of tumors and the expression level is associated with malignant progression [161]. In one study, podoplanin expression was observed in 83% and 27% of GBM and medulloblastoma cases, respectively [162]. The surrounding brain parenchyma was not stained.

The binding affinity of NZ-1-(scdsFv)-PE38KDEL is lower than that of NZ-1 antibody for podoplanin peptide, measuring 8.0 × 10^−8 ^M and 3.9 × 10^−10 ^M, respectively [110]. NZ-1-(scdsFv)-PE38KDEL retains 33–98% of its activity after incubation at 37°C for 3 days. In vitro, NZ-1-(scdsFv)-PE38KDEL is highly cytotoxic, with IC_50_ of 1.6–29 ng/mL for GBM and medulloblastoma cell lines. Intratumoral injection (0.3 mg/kg) every other day with a total of three injections resulted in tumor-growth delay in the subcutaneous tumor models of D2159MG and D283MED cells, and no toxicity-related deaths or adverse effects were observed in the treated animals. When given (0.1–3.0 *μ*g/100 *μ*L) to NSG mice through an Alzet pump over a three-day period, toxicity-associated mortality was also not observed and mouse survival increased by 41% compared to the controls.

#### 3.4.4. DmAb14 m-(scFv)-PE38KDEL (DmAb14 m-IT) Targeting Gangliosides 3′-isoLM1 and 3′,6′-isoLD1

DmAb14 m-IT is generated by fusing the scFv from mutated antibody DmAb14 (DmAb14-86184) to PE38KDEL [111]. DmAb14 is an IgM antibody, specific for 3′-isoLM1 and 3′,6′-isoLD1 [163]. The ganglioside 3′-isoLM1 is shown to be expressed in 48% and 3′,6′-isoLD1 in 68% of high-grade gliomas [164]. Their expression is restricted to human adult brain tissue [164]. Piao et al. have shown that *K*_d_ of DmAb14 m-IT for 3′-isoLM1 and 3′,6′-isoLD1 is 2.6 × 10^−9 ^M [111]. IC_50_ was determined to be 80 ng/mL (1194 pM) for the D54MG cells, 5 ng/mL (75 pM) for the D336MG cells, and 0.5 ng/mL (7.5 pM) for the D2224MG cells. There was no cytotoxicity on ganglioside-negative HEK293 cells. No animal studies have been reported.

#### 3.4.5. IT-87 Targeting BT32/A6

IT-87 is generated by fusing two complementarity-determining regions (VLCDR1 and VHCDR3) through a cognate framework region (VHFR2) to DT388 [112]. The sequence of VLCDR1-VHFR2-VHCDR3 mimetic fragment is from a human mAb specific to the cell cycle-independent glioma surface antigen BT32/A6 (US Patent number 5639863) [165, 166]. The studies by Zhou et al. demonstrated that IT-87 killed 90% of BT32/A6-expressing U87 cells at concentrations ≥ 10^−7 ^M, but not BT32/A6-negative Raji cells with concentrations even up to 10^−6 ^M [112]. In SCID mice bearing both U87 and Raji tumors, a 20-day treatment regimen (i.p., 300 *μ*g/day) beginning 6 days after inoculation of tumor cells inhibited the U87 tumor growth, but not the Raji tumor growth. Imaging studies showed that IT-87 could penetrate into the U87 tumors within 1-2 h, accumulate in the tumor core 3-4 h later, and distribute within almost the entire tumor 6 h later after intraperitoneal injection (150 *μ*g/mouse).

## 4. Specific Challenges for RIT Therapy of GBM

Reviewing various preclinical studies and clinical trials, there is no doubt that RITs are one of the most promising modalities for GBM therapy. However, it is still challenging to move RIT therapy to clinical practice, although a group of RITs have completed their Phase I/II studies or are undergoing clinical trials. Regarding RIT therapy for cancers arising from peripheral organs, top challenges include vascular leak syndrome, hepatotoxicity, the RITs' immunogenicity, and their low penetration capabilities. Consequently, developing an efficient drug delivery technique is more urgent and critical for GBM therapy. Some other critical issues in achieving effective treatment of GBM with RITs include neurological toxicity due to their target expression in normal brain tissues, the low surface expression and expression heterogeneity of targeted antigens, and poor capability of RIT penetration.

### 4.1. Technical Issues of CED

Delivery of high-molecular-weight therapeutic proteins such as RITs to intracranial tumors is extremely challenging because of the blood-brain barrier [167, 168]. CED is primarily designed to bypass the blood-brain barrier, in which a drug is delivered directly to the brain tumor through one or more catheters and circulated throughout with the use of pressure gradients. The catheters are placed either into the tumor, the tumor resection cavity, or the cavity wall. A significant advantage of CED is its potential to deliver high concentrations of RITs to the tumor site, accompanied by reduced risk of systemic toxicity. This potential has been clearly demonstrated in both preclinical studies and clinical trials. However, several technical issues have still limited its successful use in patients. Improper catheter placement, insufficient infusion, and infusate reflux have been observed in a considerable number of patients. Some clinical trials failed just due to these technical issues of CED. The other factors influencing CED success come from tumor itself. GBM tissue is characterized by high heterogeneity (pseudopalisading necrosis, cellular heterogeneity, hemorrhage, fibrin clot formation, etc.), which makes consistent drug distribution difficult. The stromal stiffening, high interstitial pressure, and heterogeneous pressure gradient further affect the delivery and distribution of RITs. Because there are many excellent reviews on CED, this review will not discuss these issues.

### 4.2. Immunogenicity

The immunogenicity of a RIT can be induced by either the scFv component or the toxin moiety. Because the immunogenicity of a mAb mainly exists in its Fc region and this region has been removed when constructing RITs, the scFv component possesses only a very weak immunogenicity when it is derived from a murine mAb. Therefore, the immunogenicity is mainly induced by the toxin moiety in most patients, which presents neutralizing antibody induction, leading to loss of the RIT's efficacy, thus limiting repeated use of RITs.

Several strategies have been attempted to minimize this issue by either reducing the immunogenicity of the toxin moiety or suppressing the patients' immune system. Immunosuppressive drugs concurrent with RIT therapy have been demonstrated to be less effective in preventing, delaying, or limiting the production of neutralizing antibodies in patients. Pegylation is a common strategy to reduce the protein immunogenicity but is found to significantly diminish the efficacy of RITs, possibly due to the blockage of the RIT scFv-antigen binding and/or improper conjugation between polyethylene glycol polymer and RITs. Genetic elimination of immunodominant T- and/or B-cell epitopes is a strategy under studies and showed reduced production of neutralizing antibodies to certain degree in animals, but this approach seems to be decreasing the activity of the toxin moiety as well, and its effectiveness in patients is still unknown. The immunogenicity issue has also been addressed by developing RITs with human endogenous cytotoxic enzymes such as RNase, granzyme B, and death-associated protein kinase 2. In general, studies are very limited and the activity of human endogenous cytotoxic enzymes is far less efficient than that of DT and PE.

### 4.3. Off-Target Neurological Toxicity

The neurological toxicity is induced by two reasons. One is that most targeted antigens or receptors are expressed not only on the tumor cells but also on normal tissues though at a much lower level. Clinical trials have demonstrated that binding with the antigens or receptors on normal brain cells could result in dose-limiting neurological toxicity. It is highly preferable to target an antigen that is tumor-specific. Another cause of neurological toxicity is infusate reflux and its distribution to normal brain tissue [169]. Reflux occurs when the pressure gradient between cannula and tumor region equalizes and results in the loss of drug flow into the target mass. Tissue disruption at the tip of the cannula, infusion rate, and cannula diameter also contributes to the infusate reflux. Postprocedural imaging has been suggested to track the delivery, for example, by mixing the RITs with an imaging agent [65, 170]. This approach may help confirm accurate cannula placement, monitor whether the drug spreads through the tumor or leaks to normal tissues, and allow real-time adjustments.

### 4.4. Factors Influencing the Inherent Activity of RITs

Several cofactors determine the inherent activity of a RIT. As discussed above, the molecular weight, valency, and structure of a RIT have the most important and direct effects on binding, penetrating, targeting, and cell-killing efficiency. Similar to mAbs, penetration of RITs into tumors is through a process of diffusion, which is affected by their molecular size and binding affinity as well as by the properties of antigens such as density, distribution, and internalization rate. Decreased penetration rate following binding with antigens is referred to as “binding-site barrier” [171–173]. Smaller RITs and those with higher binding affinity generally have better penetration capabilities. Some studies have shown that the binding-site barrier could be overcome by increasing the dose, but then off-target toxicity will increase as well. In this respect, increasing the stability of a RIT by optimizing its structure offers an approach to enhance the penetration and accumulation of RITs in tumors.

One more important issue influencing the efficacy of RITs is the antigen or receptor heterogeneity in cancer [174, 175]. Preclinical studies have established the high cytotoxic effects of RITs on brain tumor cell lines and tumor xenografts, but the antigens of the human primary and metastatic tumors are not homogenous. They are always variable in density or structure. An RIT may kill a population of GBM cells with high expression of a specific antigen or receptor, but it may subsequently has less or no killing effect on those without expression. An interesting finding in some studies in immunocompetent mice or rats is the involvement of immune response to tumors following RIT therapy. For example, treatment with an EGFRvIII-specific RIT can eliminate not only the cells with EGFRvIII but also those without EGFRvIII [57–59]. The induced immune response following RIT therapy is an exciting finding but requires more studies for evaluation.

## 5. Conclusion

RITs possess various superior properties over mAbs and traditional chemotherapeutics. Their high specificity, extreme potency, and nonoverlapping killing mechanisms and toxicity profiles to other agents may also be beneficial for RITs as part of a combined treatment with other agents. RITs are also particularly appropriate for patients with recurrent and widespread brain tumors that are resistant to surgery, chemotherapy, and radiotherapy. Reviewing various preclinical data and clinical findings, some RITs such as D2C7(scdsFc)-PE38, MR1-1(Fv)-PE38, and DT390-BiscFv806 are highly promising. To successfully translate RIT therapy for brain tumors, the technical issues of CED are urgent to solve, and the immunogenicity and off-target toxicity are other challenges to face.

## Figures and Tables

**Table 1:** Preclinical development of recombinant immunotoxins (RITs) for glioblastoma therapy.

Constructs of RITs	Targeting moiety	Toxin moiety	Target	Ref.
*EGFR/EGFRvIII-targeted RITs*				
D2C7-(scdsFv)-PE38KDEL (D2C7-IT)	D2C7 scFv	PE38KDEL	EGFR/EGFRvIII	[60–63]
MR1(Fv)-PE38 (MR1)	MR1 scFv	PE38	EGFR/EGFRvIII	[64–67]
MR1-1(Fv)-PE38 (MR1-1)	Mutated MR1 scFv	PE38	EGFR/EGFRvIII	[57, 66]
TGF*α*-PE38 (TP38)	Transforming growth factor *α*	PE38	EGFR	[68–71]
TGF*α*-PE40 (TP40)	Transforming growth factor *α*	PE40	EGFR	[72–74]
DAB389EGF	EGF	DAB389	EGFR	[75–80]
DT390-BiscFv806	mAb806 biscFv	DT390	EGFR/EGFRvIII	[81]
ScFv(14E1)-ETA	mAb14E scFv	PE40	EGFR/EGFRvIII	[82–85]
Anti-EGFR/LP1	Anti-EGFR scFv	Plant Luffin P1	EFGR	[86]
*IL-13R and IL-4-targeted RITs*				
IL-13PE38QQR (IL-13PE)	IL-13	PE38QQR	IL-13R	[87–89]
IL13E13K-PE38	Mutated IL-13	PE38QQR	IL-13R	[88, 90, 91]
Anti-IL-13Ra2(scFv)-PE38	Anti-IL-13Ra2 scFv	PE38	IL-13Ra2	[91, 92]
DT_390_IL13	IL-13	DT390	IL-13R	[93, 94]
IL4(38-37)-PE38KDEL (cpIL4-PE)	IL-4	PE38KDEL	IL-4R	[95, 96]
DT390-mIL4	11B11 scFv	DT390	IL-4R	[97]
*Bispecific RITs*				
DT390-ATF (DTAT)	uPA ATF	DT390	uPAR	[98–100]
DT390-IL-13-ATF (DTAT13)	uPA ATF and IL-13	DT390	uPAR/IL-13R	[100, 101]
EGFATFKDEL	uPA ATF and EGF	PE38KDEL	uPAR/EGFR	[102–104]
EGFATFKDEL7mut	uPA ATF and EGF	Mutated PE38KDEL	uPAR/EGFR	[102–104]
DTEGF13	IL-13 and EGF	DT390	IL-13R/EGFR	[105, 106]
*Others*				
8H9scFv-PE38	mAb 8H9 scFv	PE38	B7H3	[107, 108]
EphrinA1-PE38QQR	EphrinA1	PE38QQR	EphA2 receptor	[109]
NZ-1-(scdsFv)-PE38KDEL	NZ-1 scFv	PE38KDEL	Podoplanin	[110]
DmAb14m-(scFv)-PE38KDEL (DmAb14m-IT)	Mutated DmAb14 scFv	PE38KDEL	3′-isoLM1/3′,6′-isoLD1	[111]
IT-87	VLCDR1–VHFR2–VHCDR3	DT388	BT32/A6	[112]

**Table 2:** Clinical development of RITs for glioblastoma therapy.

RITs	Clinical trials	Status	Outcome and side effects	Ref.
D2C7(scdsFv)-PE38 (D2C7-IT)	Phase I/II	Ongoing	N/A	NCT02303678
IL-4(38-37)-PE38KDEL (cpIL4-PE)	Phase I/II	Ongoing	MS^*∗*^: 4.7 months; six-month survival: 36%	[113–115]
			Headache, seizure, weakness, dysphasia, hydrocephalus	NCT00014677
IL13-PE38QQR (IL-13PE)	Phase I/II/III	Not active	MS: 42.7 weeks in phase II and 36.4 weeks in phase III	[116–119]
			Headache, dysphasia, seizure, weakness, pulmonary embolism	
TGF*α*-PE38 (TP38)	Phase I	Discontinued	MS: 28 weeks (95% CI, 4.1–45.1)	[58, 59, 120, 121]
			Grade 3 hemiparesis, grade 4 fatigue, headache, dysphasia	
			Less effective in >80% intracranial infusions	
DAB389EGF	Phase I/II	Discontinued	N/A	[122]
MR1-1(Fv)-PE38 (MR1-1)	Phase I	Discontinued	Low accrual	[123]

^*∗*^MS, median survival.
